# Optimal infiltration depth threshold for low-temperature plasma ablation in fungal keratitis

**DOI:** 10.1186/s12348-025-00501-w

**Published:** 2025-07-01

**Authors:** Zhengwei Yang, Miaomiao Liu, Guihua Yang, Lijin Wen, Juan Yang, Hanqiao Li, Zhiwen Xie, Xie Fang, Shunrong Luo, Xianwen Xiao, Yuan Lin, Huping Wu

**Affiliations:** 1 School of Medicine, Xiamen Eye Center and Eye Institute of Xiamen University, Xiamen, China; 2 Xiamen Clinical Research Center for Eye Diseases, Xiamen, Fujian China; 3 Xiamen Key Laboratory of Ophthalmology, Xiamen, Fujian China; 4 Fujian Key Laboratory of Corneal & Ocular Surface Diseases, Xiamen, Fujian China; 5 Xiamen Key Laboratory of Corneal & Ocular Surface Diseases, Xiamen, Fujian China; 6 Translational Medicine Institute of Xiamen Eye Center of Xiamen University, Xiamen, Fujian China; 7 Department of Medical Ultrasonics, The third affiliated hospital of Zhengzhou university, Zhengzhou, Henan Province China; 8 Department of Ophthalmology, Fujian Provincial Key Laboratory of Ophthalmology and Visual Science; Fujian Engineering and Research Center of Eye Regenerative Medicine; School of Medicine, Xiang’an Hospital of Xiamen University; Eye Institute of Xiamen University, Xiamen University, Xiamen, Fujian Province China

**Keywords:** Fungal keratitis, Low-temperature plasma ablation, Prognostic factors, Infiltration depth, Clinical outcomes

## Abstract

**Purpose:**

To identify prognostic factors and their optimal thresholds influencing the treatment outcome of low-temperature plasma ablation therapy in patients with fungal keratitis (FK).

**Methods:**

The clinical information of fifty-one patients with FK treated with low-temperature plasma ablation at Xiamen Eye Center from 2018 to 2024 was retrospectively analyzed. Patients were categorized into Responder and Non-Responder groups based on their response to treatment: complete/partial healing (Responder group) versus disease progression or need for additional surgery (Non-Responder group). Differences in demographic and clinical characteristics between the two groups were compared. Additionally, exact univariate and multivariate logistic regression were performed to identify prognostic factors. Lastly, receiver operating characteristic (ROC) curve analysis was utilized to determine the cut-off value for significant prognostic factors.

**Results:**

Among the 51 patients, 37 were classified in the Responder group and 14 in the Non-Responder group. Univariate analysis revealed significant differences in the presence of hypopyon (*p* = 0.038), ulcer size (*p* = 0.002), infiltration depth (*p* = 0.001), and paracentral ulcer location (*p* = 0.030) between the groups. Multivariate analysis identified infiltration depth (adjusted odds ratio [aOR] = 1.41, 95% CI: 1.05–1.91, *p* = 0.024) as the sole significant independent prognostic factor. ROC analysis demonstrated excellent discrimination ability for infiltration depth, with an area under the curve of 0.966. Finally, the optimal threshold for infiltration depth was determined to be 0.48, exhibiting a sensitivity of 92.86% and specificity of 91.89%.

**Conclusions:**

Low-temperature plasma ablation represents an effective treatment for FK, with infiltration depth serving as a crucial prognostic indicator. The identified threshold provides valuable guidance for patient selection. Nonetheless, larger prospective studies are warranted to validate these findings.

**Supplementary Information:**

The online version contains supplementary material available at 10.1186/s12348-025-00501-w.

## Introduction

Fungal keratitis (FK), a severe corneal infection causing vision loss if untreated, accounts for up to 45% of infectious keratitis cases, with nearly one million annual cases globally, particularly in tropical regions [1]. Current treatment options involve topical and systemic antifungal drugs. However, their efficacy is limited by broad-spectrum activity and the emergence of antifungal resistance [2]. Severe cases may require surgical interventions such as conjunctival flap coverage or therapeutic keratoplasty. However, the former may result in severe loss of corneal transparency, while the latter may elicit graft rejection, recurrence of infection, and secondary glaucoma [3]. Therefore, there is a pressing need to identify effective and safe treatments for FK to mitigate vision-related adverse effects in FK patients.

As is well documented, low-temperature plasma has demonstrated significant promise in biomedical applications owing to its ability to perform precise surgical cutting, sealing, and hemostasis while maintaining a low temperature of 25–28 °C [4]. The low-temperature plasma ablation system (PLA-700) generates a plasma layer by stimulating a saline electrolyte through a 100 kHz radio frequency field to achieve a controlled ablation depth of approximately 50 μm [5]. In the treatment of FK, this technique can effectively remove superficial corneal layers and fungal components, enhance topical antifungal drug penetration, and improve therapeutic efficacy [6]. Noteworthily, earlier studies have established its efficacy as an adjuvant therapy, with the low-temperature plasma group associated with higher rates of complete healing, faster recovery, and superior visual acuity compared to conventional therapy [7, 8].

Despite the promising results of low-temperature plasma ablation therapy for the management of FK, factors influencing treatment outcomes and prognosis remain elusive. Previous studies have identified several potential prognostic factors for FK, including the causative fungal species, the size and depth of the corneal lesion, the presence of hypopyon, and the timing of treatment initiation [9]. However, these factors have not been systematically investigated in the context of low-temperature plasma ablation therapy for FK, with optimal thresholds remaining to be elucidated.

Thus, this study aimed to identify prognostic factors and their optimal thresholds that influence the treatment outcomes of low-temperature plasma ablation therapy in FK patients to improve patient selection, optimize treatment strategies, and ultimately improve the effectiveness and safety of this therapeutic modality.

## Methods

This retrospective study was conducted at the Xiamen Eye Center and Eye Institute of Xiamen University from November 2018 to January 2024. It was approved by the Ethics Committee of Xiamen Eye Center and Eye Institute of Xiamen University (XMYKZK-KY-2024-068) and was performed in accordance with the tenets of the Declaration of Helsinki. Written informed consent was obtained from all patients prior to the surgical intervention. The medical records of patients diagnosed with FK who underwent low-temperature plasma ablation therapy were reviewed. The inclusion criteria were as follows: (1) clinically diagnosed FK confirmed by in vivo confocal microscopy and corneal scraping with culture, (2) age ≥ 18 years, (3) treatment with low-temperature plasma ablation therapy, and (4) poorly controlled patients with at least one week of medical treatment. The exclusion criteria encompassed a history of corneal surgery, full-thickness or perforated corneal lesions, coexisting ocular infections, systemic diseases including diabetes mellitus and autoimmune diseases, or incomplete medical records.

### Ophthalmologic examination

A total of 51 patients (51 eyes) met the inclusion and exclusion criteria. Demographic and clinical data, comprising age, gender, duration of symptoms at presentation, medical history, history of ocular trauma, and length of hospital stay, were extracted from electronic medical records. The included patients underwent the following assessments: preoperative and postoperative best-corrected visual acuity (BCVA), intraocular pressure, slit-lamp examination, slit-lamp photography (Haag-Streit, Switzerland), anterior segment optic coherence tomography (AS-OCT; RTVue XR, Optovue Inc., USA), corneal scraping and culture, and in vivo confocal microscopy (IVCM; Heidelberg Engineering GmbH, Dossenheim, Germany).

Visual acuity was evaluated using a Snellen chart and converted to LogMAR for statistical analysis. Visual acuities of counting fingers, hand motion, light perception, and no light perception were assigned LogMAR values of 2.0, 2.3, 2.6, and 2.9, respectively [10]. During the slit-lamp evaluation, the following features were examined: satellite lesions, pseudopods, endothelial plaques, hypopyon height, ulcer location, and ulcer size (the geometric mean of the longest diameter and its perpendicular width). The depth of the infiltrate was clinically determined using a slit lamp and manually measured using anterior segment optical coherence tomography. Ulcers were defined as hyperreflective areas in the corneal stroma. To quantify ulcer thickness, one caliper arm was placed on the most anterior hyperreflective corneal surface (stromal or epithelial), whilst the second arm was placed on the posterior border of the hyperreflective area. Infiltration depth was calculated as the ratio of ulcer thickness to total corneal thickness using AS-OCT [11].

### Medical treatment

At the initial presentation, IVCM was performed to confirm the diagnosis of FK. Subsequently, corneal scrapings were collected for culture and sensitivity testing to guide antifungal therapy. Patients were initially treated with topical amphotericin B eye drops (0.25 mg/mL in sterile water for injection) every 30 min and 1% voriconazole eye drops (10 mg/mL in sterile water for injection) every 30 min. In severe cases, systemic antifungal therapy was initiated, comprising either oral itraconazole capsules or intravenous voriconazole 200 mg once daily. Antifungal agents were subsequently adjusted based on the results of fungal culture and sensitivity testing.

Patients who failed to respond to intensive medical treatment after one week were considered for alternative interventions. Those who were either unwilling to undergo corneal keratoplasty or deemed unsuitable candidates for conjunctival flap coverage were offered low-temperature plasma ablation therapy.

### Low-temperature plasma ablation treatment

Low-temperature plasma ablation was performed under a peribulbar block and strict aseptic conditions. After the eye was opened, the PLA-700 system (Chengdu Mechanical and Electronic Technology Co., Ltd.) was utilized, employing 0.9% NaCl as the conductive medium. The active electrode at the handpiece tip generated plasma within a 50 μm ablation depth at an average temperature of 25–28 °C. The ablation function was activated with the power setting adjusted to 30 W, and the treatment duration lasted approximately 30 to 60s, depending on the extent of the corneal ulcer. The handpiece was manipulated vertically, making minimal contact with the corneal surface, with horizontal and vertical rotations marginally exceeding the ulcer boundaries. Post-operatively, patients continued to receive antifungal treatment based on the sensitivity results.

### Follow-up and outcomes

Following surgical and medical therapy, patients were subjected to follow-up examinations every two weeks during the first postoperative month and monthly thereafter. At each visit, BCVA, corneal ulcer size, ulcer depth, hypopyon height, and adverse events were documented. At the 3-month follow-up, patients were categorized into Responder and Non-Responder groups based on treatment outcomes.

The Responder group consisted of patients who achieved complete or partial healing of corneal lesions. Complete healing was defined as the resolution of the corneal ulcer, negative fluorescein staining, and absence of hypopyon. Partial healing was characterized by a reduction in corneal ulcer area exceeding one-third, accompanied by a decrease or disappearance of hypopyon. The Non-Responder group included patients exhibiting progressive ulcer size, increased infiltration depth, corneal perforation, hypopyon greater than 2 mm, or those requiring additional surgical interventions shortly after combined treatment.

### Statistical analysis

Continuous variables were expressed as mean ± standard deviation or median (interquartile range) and compared using the Student’s t-test or Mann-Whitney U test. Categorical variables were presented as frequencies and percentages and compared using the chi-square or Fisher’s exact test. Exact logistic regression analyses were performed to identify prognostic factors associated with treatment failure. Variables with *P* < 0.1 in the univariate analysis were incorporated in the multivariate model. A P-value < 0.05 was considered statistically significant. Statistical analyses were performed using SPSS version 23.0 (IBM Corp., Armonk, NY, USA), and exact logistic regression was carried out using Stata 16 (Stata Corp. 2019, Stata Statistical Software: Release 16, Stata Corp LLC).

Receiver operating characteristic (ROC) analysis was conducted using MedCalc software (MedCalc Inc., Mariakerke, Belgium) to establish optimal thresholds for prognostic factors by evaluating the area under the curve (AUC) to assess its discrimination ability. Sensitivity and specificity were calculated to identify the most suitable cut-off values. All statistical tests were two-tailed, with a p-value of less than 0.05 considered statistically significant.

## Results

A total of 51 patients with FK were recruited to receive low-temperature plasma ablation treatment. Among them, 26 (50.98%) were men, and 25 were women, with an average age of 53.33 ± 13.52 years. Based on the treatment outcomes, 37 patients were classified in the Responder group, whereas the remaining 14 patients were assigned to the Non-Responder group. The demographic and baseline clinical characteristics are presented in Table 1. Comparison between the two groups revealed statistically significant differences in the presence of hypopyon (*p* = 0.02), ulcer size (*p* < 0.001), infiltration depth (*p* < 0.001), and ulcer location (*p* = 0.02). To further illustrate the therapeutic outcomes of low-temperature plasma ablation, representative slit-lamp images comparing baseline and 3-month post-treatment outcomes are displayed in Supplementary Fig. S1 and Fig. S2. Visual outcomes, as listed in Table 2, uncovered significant differences in preoperative (*p* = 0.005) and postoperative BCVA (*p* < 0.001) between the two groups. Additionally, significant differences were observed in the preoperative and postoperative BCVA of patients in the Responder group (*p* < 0.001), whereas no significant differences were noted in the Non-Responder group (*p* = 0.95). The species distribution detailed in Supplementary TableS1 uncovered that the positive fungal culture rate was 37.25% (19/51), with Fusarium (13.73%) and Aspergillus fumigatus (5.88%) as the predominant isolates.

**Table 1:** Baseline characteristics

Parameters	Total	Responder group	Non-Responder group	*p*-value
Participants (n)	51	37	14	
Age, mean (SD), y	53.33 (13.52)	51.65 (13.77)	57.79 (12.18)	0.26
Gender, male (%)	26 (50.98%)	20 (54.05%)	6 (42.86%)	0.54
Duration of symptoms at presentation, mean (SD), d	16.75 (25.54)	13.50 (11.21)	25.38 (45.28)	0.48
Receipt of prior antifungal therapy	5 (9.80%)	3 (8.11%)	2 (14.29%)	0.89
Satellite lesions	8 (15.69%)	6 (16.22%)	2 (14.29%)	0.79
Pseudopods	21 (41.18%)	14 (37.84%)	7 (50.00%)	0.43
Endothelial plaques	5 (9.80%)	2 (5.41%)	3 (21.43%)	0.23
Hypopyon, mean (SD), mm	0.20 (0.61)	0.08 (0.36)	0.50 (0.96)	**0.02**
Ulcer size, mean (SD), mm	4.38 (12.4)	4.01 (0.95)	5.36 (1.39)	**<0.001**
Infiltration depth, mean (SD)	0.47 (0.14)	0.40 (0.07)	0.65 (0.11)	**<0.001**
Ulcer location				
Central	24 (47.06%)	13 (35.14%)	11 (78.57%)	**0.02**
Paracentral	23 (45.10%)	20 (54.05%)	3 (21.43%)	
Peripheral	4 (7.84%)	4 (10.81%)	0 (0)	
Length of hospital stay, mean (SD), d	10.16 (5.37)	10.06 (5.01)	10.43 (6.38)	0.88

*p*-value refers to the differences between the Responder group and Non-Responder group, SD: standard deviation, d: days, mm: millimetre, significant differences in bold

**Table 2:** Comparison of BCVA at 3 months postoperatively

Time	Responder group	Non-Responder group	*p*-value
Preoperative BCVA, mean (SD)	1.25 (0.78)	1.88 (0.67)	**0.005**
Postoperative BCVA, mean (SD)	0.63 (0.58)	1.80 (0.86)	**<0.001**
***p*** **-value**	**<0.001**	0.95	
Mann-Whitney test			

Exact univariate and multivariate logistic regressions incorporating patients’ lesion signs were performed to identify prognostic factors between the Responder and Non-Responder groups (Table 3). In the univariate analysis, the presence of hypopyon (OR = 7.00, 95% CI: 1.11 to 43.95, p = 0.038), ulcer size (OR = 2.83, 95% CI: 1.44 to 5.55, p = 0.002), infiltration depth (OR = 1.28, 95% CI: 1.11 to 1.49, p = 0.001), and paracentral ulcer location (OR = 0.18, 95% CI: 0.03 to 0.88, p = 0.030) were significantly associated with treatment outcomes. In the multivariate analysis, only infiltration depth (adjusted odds ratio [aOR] = 1.41, 95% CI: 1.05–1.91, p = 0.024) emerged as a significant independent risk factor for treatment outcomes.

**Table 3:** Univariate and multivariate logistics regression analysis of factors associated with low-temperature plasma ablation therapy for FK

Parameters	Univariable logistic regression			Multivariable logistic regression		
	OR	95% CI	*p*-value	OR	95% CI	*p*-value
Age	1.04	0.99 to 1.10	0.153			
Gender						
Female	ref	ref	ref			
Male	0.64	0.18 to 2.20	0.477			
Duration of symptoms at presentation	1.02	0.99 to 1.05	0.247			
Receipt of prior antifungal therapy						
No	ref	ref	ref			
Yes	1.89	0.28 to 12.71	0.513			
Presence of satellite lesions						
No	ref	ref	ref			
Yes	0.86	0.15 to 4.87	0.866			
Presence of pseudopods						
No						
Yes	1.64	0.48 to 5.68	0.433			
Presence of endothelial plaques						
No	ref	ref	ref			
Yes	4.77	0.70 to 32.33	0.109			
Hypopyon	7.00	1.11 to 43.95	**0.038**	12.84	0.01 to 123.7	0.585
Ulcer size	2.83	1.44 to 5.55	**0.002**	1.03	0.13 to 8.05	0.979
Infiltration depth	1.28	1.11 to 1.49	**0.001**	1.41	1.05 to 1.91	**0.024**
Ulcer location						
Central	ref	ref	ref			
Paracentral	0.18	0.03 to 0.88	**0.030**	0.07	0.00 to 1.90	0.114
Peripheral	0.25	0.00 to 2.23	0.233			
Length of hospital stay	1.01	0.90 to 1.14	0.824			

OR: odds ratio, CI: Confidence Interval, ref: reference, significant differences in bold

**Fig. 1:**
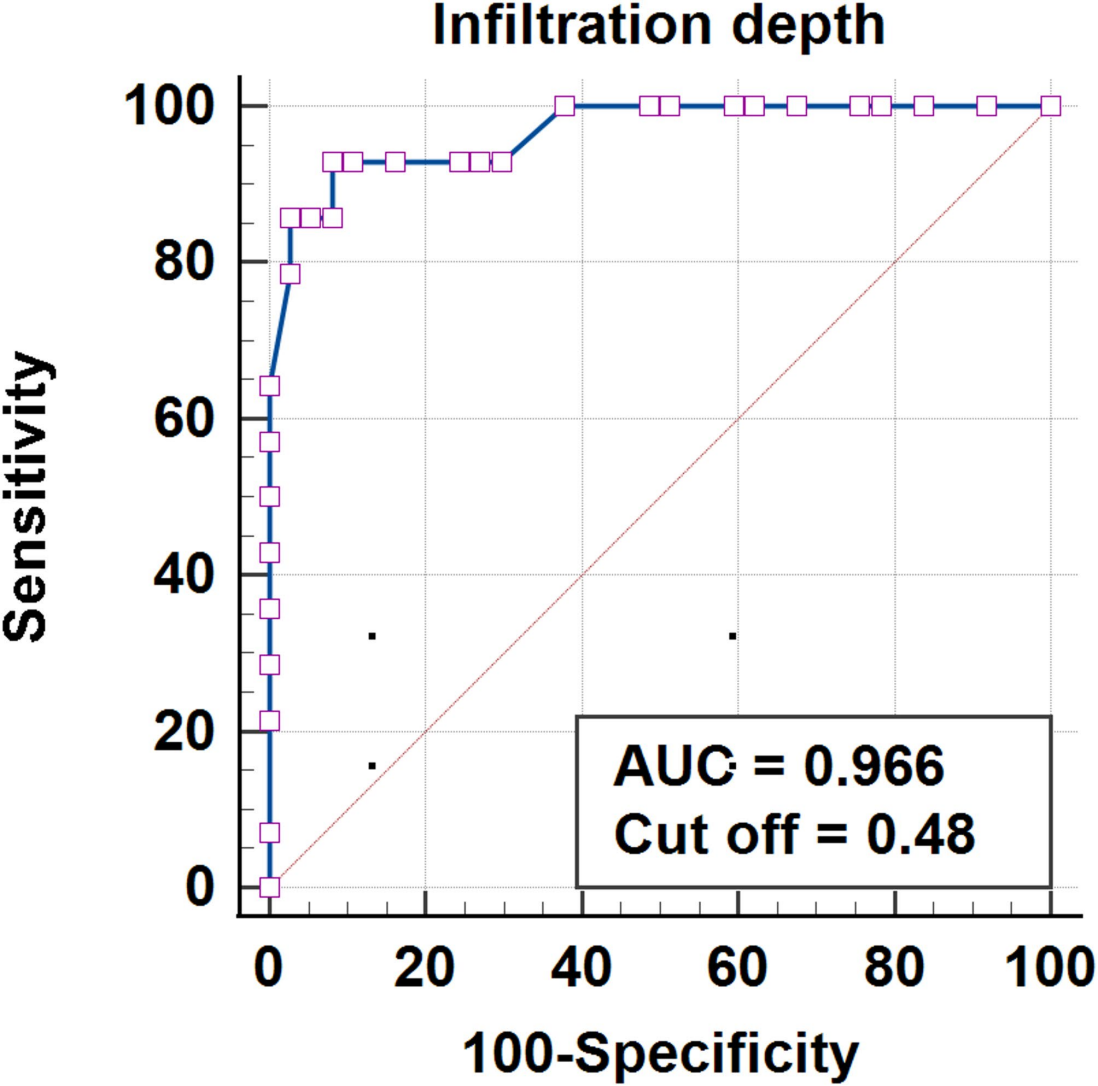
ROC curve for infiltration depth in predicting treatment outcomes. The AUC is 0.966, with an optimal cut-off value of 0.48

Next, ROC curve analysis was employed to determine the optimal cut-off values for the significant prognostic factors identified in the multivariate analysis. The AUC for the infiltration depth was 0.966 (95% CI: 0.873–0.997, *p* < 0.001), indicating favorable discrimination ability. The optimal cut-off value for the infiltration depth was 0.48, with a sensitivity of 92.86% and specificity of 91.89% (Fig. 1).

## Discussion

This study investigated prognostic factors and optimal thresholds influencing the outcomes of low-temperature plasma ablation therapy for FK. Our findings demonstrated significant differences between the Responder and Non-Responder groups in terms of hypopyon, ulcer size, infiltration depth, and ulcer location. Notably, infiltration depth emerged as an independent prognostic factor, with a strong correlation with treatment outcomes, as reflected by a high area under the curve (AUC) value of 0.966 in ROC analysis. The optimal threshold for infiltration depth was determined to be 0.48, providing high sensitivity and specificity in predicting treatment outcomes. These results collectively suggest that low-temperature plasma ablation is a promising adjunctive therapy for FK, potentially improving visual acuity when combined with antifungal treatments.

A previous study outlined that low-temperature plasma ablation could be an effective adjuvant therapy to topical antifungal medications for patients with mild to moderate FK [7]. In that study, the success rate for FK treated with low-temperature plasma ablation surgery was 73.3%, which closely aligned with our surgical success rate of 72.5% (37/51). Multivariate analysis identified infiltration depth as the most significant independent prognostic factor, with ROC curve analysis demonstrating excellent discriminatory ability (AUC = 0.966, *p* < 0.001). The optimal threshold for infiltration depth was determined to be 0.48, providing impressive diagnostic performance with a sensitivity of 92.86% and specificity of 91.89%. These results not only confirm the therapeutic efficacy of low-temperature plasma ablation but also highlight the relevance of infiltration depth as a crucial prognostic indicator. Furthermore, these findings provide clinicians with a novel quantitative tool for objectively stratifying low-temperature plasma ablation candidates, offering actionable guidance to optimize clinical decision-making and patient selection. Given the limited research on this therapy, these insights hold substantial reference value in establishing specific criteria for its application, thereby improving treatment strategies and outcomes for patients with FK. Nonetheless, further prospective studies are necessitated to validate the safety and efficacy of infiltration depth in the treatment of FK with low-temperature plasma ablation.

Of note, this study revealed that hypopyon, ulcer size, and ulcer location were significantly associated with treatment outcomes in the univariate analysis. Hypopyon has consistently been recognized as a significant prognostic factor for treatment outcomes in FK, given that other studies have documented that its presence often correlates with poorer prognosis due to increased inflammation and severity of infection [9, 12]. Similarly, larger ulcer sizes have been associated with more severe disease and worse visual outcomes, validating the findings of the univariate analysis in this study that larger ulcers are linked to ineffective treatment [13, 14]. Ulcer location is also a critical factor, with paracentral ulcers exerting a more pronounced effect on visual acuity compared to peripheral ulcers [15, 16], consistent with the findings of the current study that ulcer location was identified as a key factor in the univariate analysis. When comparing low-temperature plasma ablation therapy to traditional treatments, this study suggests that surgical intervention offers comparable or superior outcomes with fewer complications. Traditional surgical methods, such as conjunctival flap covering and keratoplasty, may lead to loss of corneal transparency and graft rejection, whereas low-temperature plasma ablation provides a less invasive alternative with promising recovery rates and improved visual acuity [7]. This positions low-temperature plasma ablation as a promising adjunctive treatment for FK, with the potential to enhance patient outcomes while minimizing the risk of adverse effects.

According to a previous study, corneal scraping tends to result in poorer BCVA outcomes compared to low-temperature plasma ablation [17]. This discrepancy may be attributed to low-temperature plasma ablation being less invasive and causing less postoperative inflammation. The mechanism of low-temperature plasma ablation in the treatment of FK involves a sophisticated interaction between physical and biological processes that enhance therapeutic outcomes [8]. During the procedure, the saline medium is ablated by low-temperature plasma, resulting in the formation of a thin layer of particles around the electrode. In turn, this layer disrupts the organic molecular chains in the tissue, effectively separating the molecules from the cells of the target tissue [18]. A significant amount of plasma energy is delivered to the tissue at relatively low temperatures, leading to organic bond breakage and disruption of the molecular structure, thereby achieving tissue ablation [19]. Additionally, the thermal and electromagnetic properties of the plasma layer potentially induce localized microenvironmental alterations that may disrupt fungal cell membranes and metabolic processes, complementing the mechanical removal of infected tissue [20]. Thus, this surgical approach enables the targeted removal of superficial epithelial layers and fungal elements from the corneal surface. Besides, this precise ablation mechanism may enhance the penetration of topical antifungal medications by disrupting the physical barriers that typically limit drug absorption [7]. Consequently, low-temperature plasma ablation represents a promising adjunctive treatment for FK, with the potential to improve patient outcomes while minimizing adverse effects.

Nevertheless, some limitations of this study cannot be overlooked. To begin, the retrospective nature of the study limited the ability to establish causality and may introduce selection bias. Secondly, the relatively small sample size restricted the generalizability of the findings, given that it may not adequately represent the broader population affected by FK. Furthermore, the retrospective nature and limited sample size of this study restricted our ability to control for consistency of baseline data, including preoperative BCVA. Additionally, reliance on medical records for data collection may result in incomplete or inaccurate documentation of clinical parameters, thereby potentially introducing bias. Moreover, despite a culture-positive rate of 37.25%, the low productivity highlights the need for molecular diagnostics in future studies to improve pathogen identification. Finally, the exclusion of patients with systemic diseases or a history of prior ophthalmic surgery may limit the applicability of the results to more complex clinical scenarios. Therefore, further studies with larger sample sizes and standardized protocols are necessitated to facilitate the application of this new technique into clinical practice.

In summary, this study demonstrated that low-temperature plasma ablation therapy is an effective treatment for FK, with infiltration depth emerging as a crucial independent prognostic factor. The optimal threshold of 0.48 provides valuable guidance for patient selection and optimization of treatment outcomes. Overall, this therapy presents a promising, less invasive alternative to conventional treatments, potentially improving visual outcomes and mitigating the risk of complications. These findings highlight the relevance of infiltration depth in clinical decision-making and patient management strategies for FK, as well as emphasize the need for further prospective studies with larger sample sizes to validate these results and develop standardized treatment protocols.

## Electronic supplementary material

Below is the link to the electronic supplementary material.

Supplementary Material 1

Supplementary Material 2

## Data Availability

No datasets were generated or analysed during the current study.
