# Supplementary material for: Optimal infiltration depth threshold for low-temperature plasma ablation in fungal keratitis
Source: J Ophthalmic Inflamm Infect. 2025 Jul 1;15:52. doi: 10.1186/s12348-025-00501-w (PMC12214183; doi:10.1186/s12348-025-00501-w)
Supplement: Supplementary file 1 — Supplementary Material 1 [file 12348_2025_501_MOESM1_ESM.docx]

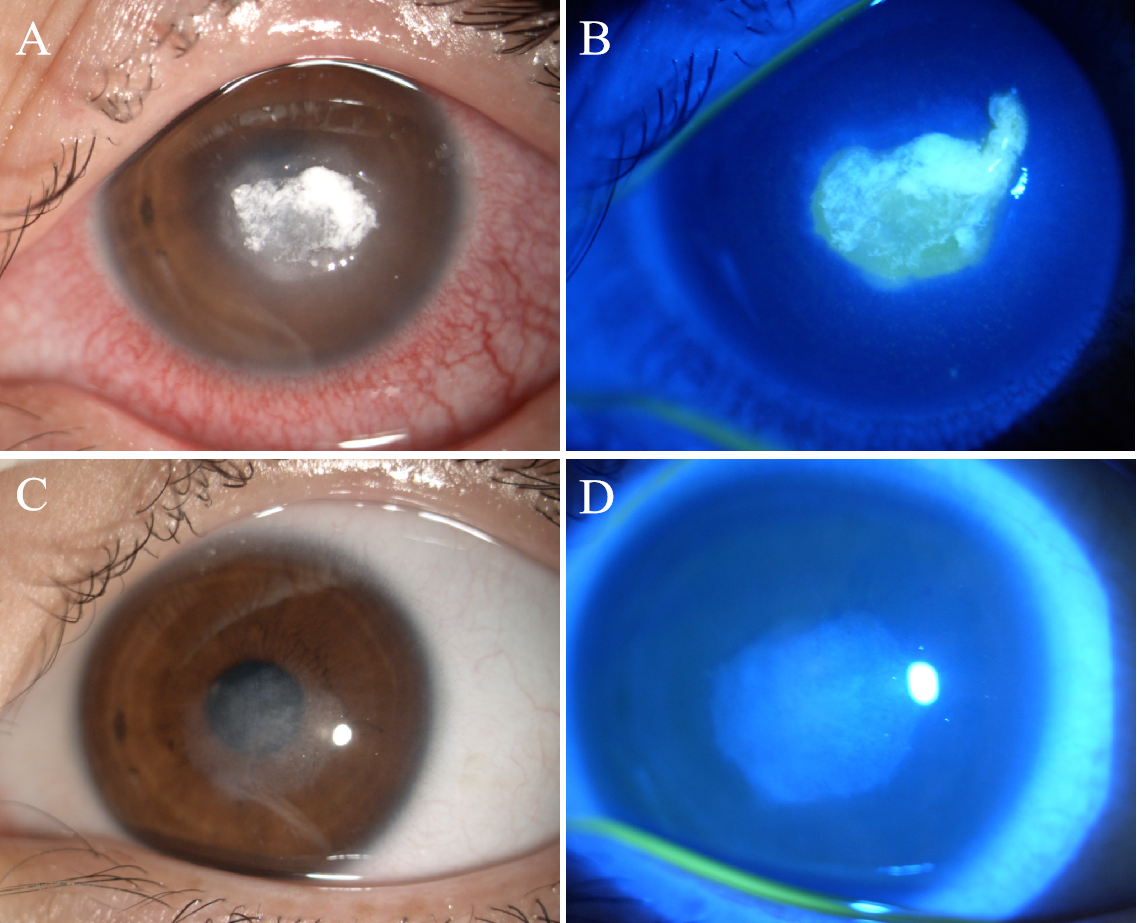


**Fig. S1** illustrates a patient from the Responder group, showing significant resolution of corneal lesions and improved corneal transparency at 3 months post-treatment. Baseline images (Panels A and B) depict a large corneal ulcer with fluorescein staining, while post-treatment images (Panels C and D) show a reduction in ulcer size and improved clarity.


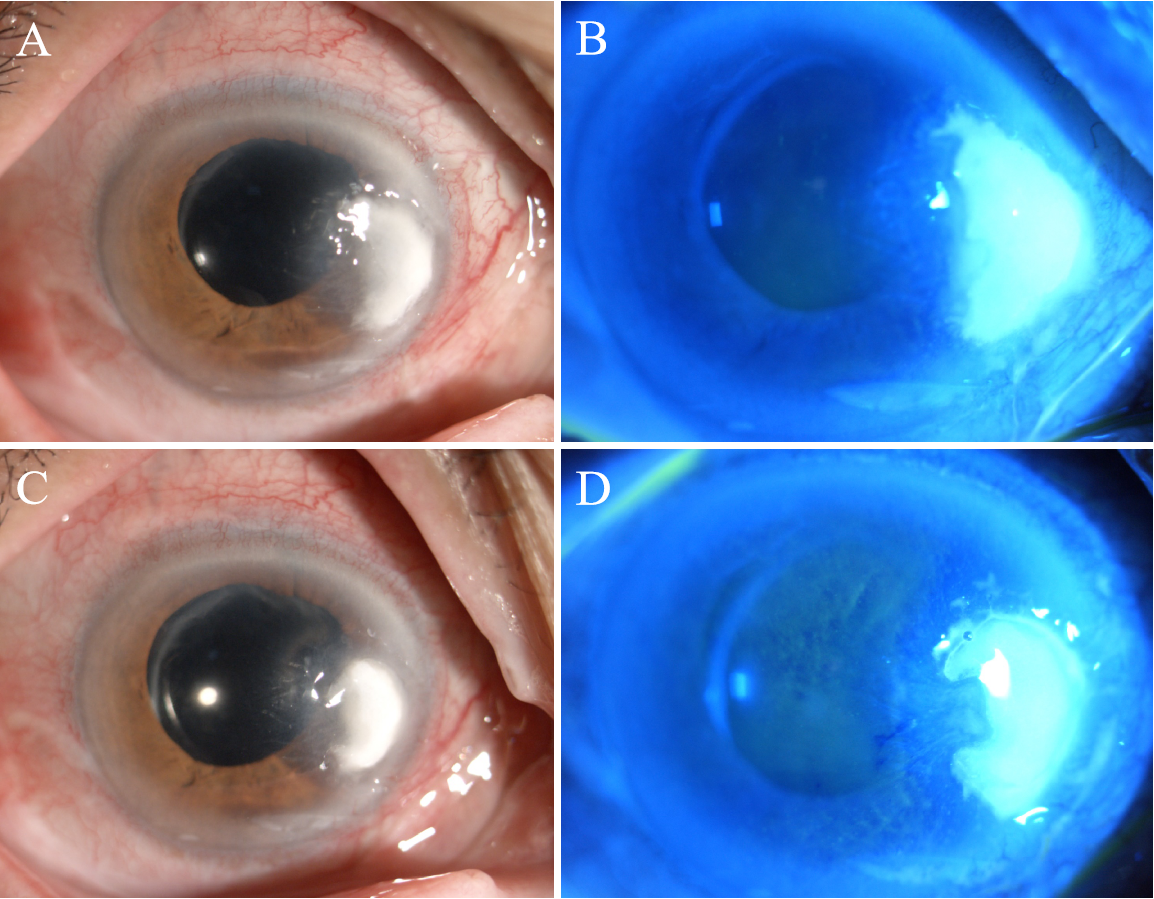


**Fig. S2** presents a patient from the Non-Responder group, highlighting progressive disease and lack of significant improvement despite 3 months of treatment. Baseline images (Panels A and B) show a thick infiltrating lesion, while post-treatment images (Panels C and D) illustrate persistent corneal opacity and no significant regression.
