# Supplementary material for: Optimal infiltration depth threshold for low-temperature plasma ablation in fungal keratitis
Source: J Ophthalmic Inflamm Infect. 2025 Jul 1;15:52. doi: 10.1186/s12348-025-00501-w (PMC12214183; doi:10.1186/s12348-025-00501-w)
Supplement: Supplementary file 2 — Supplementary Material 2 [file 12348_2025_501_MOESM2_ESM.docx]

| Supplementary Table S1. Distribution of various fungal species in patients with fungal keratitis | | |
| --- | --- | --- |
|  |  |  |
| Fungal isolates | Number | Percentage |
| **Responder group** |  |  |
| Fusarium | 5 | 9.80% |
| Aspergillus fumigatus | 3 | 5.88% |
| Dematiaceous fungi | 1 | 1.96% |
| Colletotrichum spp | 1 | 1.96% |
| Candida guilliermondii | 1 | 1.96% |
| Alternaria alternata | 1 | 1.96% |
| Curvularia Lunata | 1 | 1.96% |
| Negative isolates | 24 | 47.06% |
| **Non-Responder group** |  |  |
| Fusarium | 2 | 3.92% |
| Dematiaceous fungi | 1 | 1.96% |
| Colletotrichum spp | 1 | 1.96% |
| Scedosporium Apiospemum | 1 | 1.96% |
| Fusarium solani | 1 | 1.96% |
| Negative isolates | 8 | 15.69% |
| **All isolates** |  |  |
| Fusarium | 7 | 13.73% |
| Aspergillus fumigatus | 3 | 5.88% |
| Dematiaceous fungi | 2 | 3.92% |
| Colletotrichum spp | 2 | 3.92% |
| Candida guilliermondii | 1 | 1.96% |
| Alternaria alternata | 1 | 1.96% |
| Curvularia Lunata | 1 | 1.96% |
| Scedosporium Apiospemum | 1 | 1.96% |
| Fusarium solani | 1 | 1.96% |
| Negative isolates | 32 | 62.75% |
| Total | 51 | 100% |
